# Reverse Shoulder Arthroplasty Associated With Unnoticed Glenohumeral Dislocation: A Case Report

**DOI:** 10.7759/cureus.42769

**Published:** 2023-07-31

**Authors:** Ana Luisa Galicia-Zamalloa, Margoth Jiménez-Juárez, Andrés Pérez-Briones, David Campos-Flores

**Affiliations:** 1 Orthopaedics and Traumatology, Instituto de Seguridad y Servicios Sociales de los Trabajadores al Servicio de los Poderes del Estado de Puebla, Puebla, MEX; 2 Orthopaedics and Traumatology, Instituto de Seguridad y Servicios Sociales para Trabajadores del Estado, Puebla, MEX; 3 Orthopaedics, Instituto de Seguridad y Servicios Sociales para Trabajadores del Estado, Puebla, MEX

**Keywords:** cuff deficient shoulder, reverse shoulder arthroplasty, humeral head osteonecrosis, rotator cuff arthropathy, chronic anterior glenohumeral dislocation

## Abstract

Osteonecrosis of the humeral head is seen in rare cases of anterior shoulder dislocations. There are many different surgical procedures that have been developed to repair inveterate anterior glenohumeral dislocation. Reverse shoulder arthroplasty (RSA) is a type of surgery that has been shown to be very effective in patients with cuff tear arthropathy.

A 63-year-old female came to our service with an inveterate glenohumeral dislocation. We identified the osteonecrosis of the humeral head and decided to treat her with a reverse shoulder arthroplasty.

Osteonecrosis following a glenohumeral dislocation is a rare condition. Treatment with a reverse shoulder arthroplasty allows a fast recovery, good functional results, and a better quality of life.

## Introduction

The glenohumeral joint is the most mobile in the body, and this mobility makes it susceptible to dislocation [1]. Shoulder dislocation is the most common joint dislocation, accounting for almost 45% of all. More than 90% of shoulder dislocations are anterior dislocations, which represent 11% of all shoulder injuries [2]. The main complications of shoulder dislocation are rotator cuff tear [3], recurrence, and fractures in 25% [4]. In rare cases, osteonecrosis of the humeral head can occur after a shoulder dislocation [5].

Chronic anterior dislocation of the shoulder is common in elderly patients. The epidemiology of unnoticed glenohumeral dislocation is not well-studied. However, it is estimated that up to 10% of all shoulder dislocations go unnoticed. Although some studies have shown the functional status of the shoulder joint can be preserved after chronic anterior dislocation, there is often a loss of function, arthritis, instability, and nerve damage [6]. Surgical stabilization of the glenohumeral joint is indicated when the instability causes discomfort [7].

Many surgical procedures have been developed to repair inveterate anterior glenohumeral dislocation. Reverse shoulder arthroplasty (RSA) is a type of surgery that is very effective in patients with cuff tear arthropathy. It replaces the humeral head and glenoid fossa with prosthetic components. RSA works by stabilizing the shoulder independently of the soft tissue injuries [8-10].

We present the case of a patient with osteonecrosis associated with an unnoticed glenohumeral dislocation who was successfully treated with a reverse shoulder prosthesis.

## Case presentation

A 63-year-old woman presented to our clinic with a history of left shoulder pain and dysfunction. She reported a fall from a standing position four months earlier and she had suffered a left glenohumeral dislocation. She was initially treated conservatively with physical therapy, but her symptoms failed to improve. The patient reports continuing with functional limitation and pain 9/10 on the Visual Analog Scale (VAS) score during movements, so she decided to come to our hospital for reassessment.

This patient was presented as a healthy woman, non-alcoholic, and not on any medications. On physical examination, the patient had a limited range of motion in her left shoulder. She had 20 degrees of flexion, 20 degrees of extension, 10 degrees of abduction, and limited adduction. Internal and external rotation were abolished. The patient also had pain with shoulder movements.

**Figure 1:**
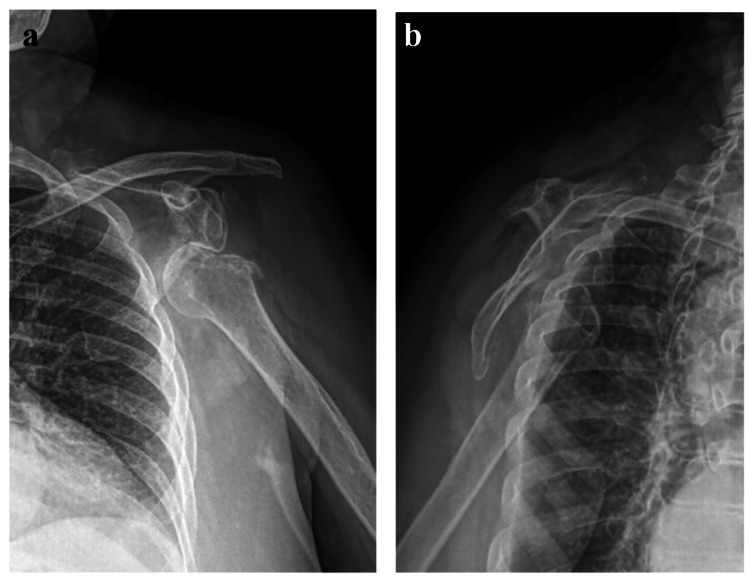
Pre-operative X-ray showing a subcoracoid subtype of anterior left shoulder dislocation showing an incongruent glenohumeral joint and an associated Hill-Sachs injury.

Radiographs of the left shoulder showed an anterior glenohumeral joint incongruity, with a bone defect at the level of the greater tuberosity, presenting areas of subchondral serpiginous sclerosis and radiolucency areas in the posterosuperior rim of the humeral head (Figure 1). 

**Figure 2:**
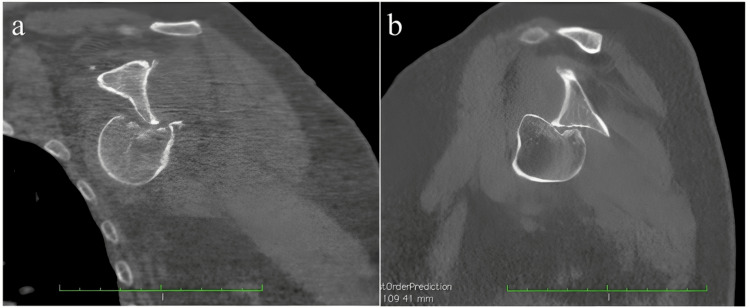
Computed tomography of the left shoulder.

Then, the axial tomography of the left shoulder in the coronal and sagittal section showed a Hill-Sachs lesion greater than 40%, changes in density corresponding to osteochondral lysis, as well as undulating sclerosis were observed, without the involvement of the glenoid cavity (Figure 2).

**Figure 3:**
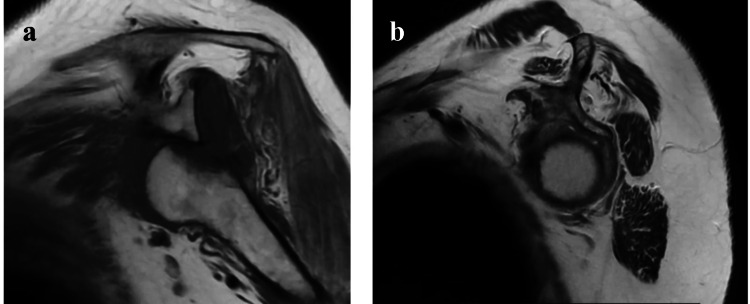
Left shoulder coronal magnetic resonance imaging (MRI) image. T2-weighted gradient echo axial MRI image showing accumulation of fat, 50% fatty muscle atrophy at the supraspinatus and infraspinatus which consists in a grade 3 of Goutallier classification and incongruent glenohumeral joint.

Magnetic resonance imaging (MRI) of the left shoulder showed severe hypotrophy of the rotator cuff, as well as fatty infiltration of the supraspinatus and infraspinatus muscles Goutallier 3, as well as changes in intensity in the humeral head with a geographical pattern (Figure 3).

Based on the patient's history, physical examination, and imaging findings, we diagnosed her with inveterate dislocation of the left shoulder with a Hill-Sachs lesion. We recommended surgery, and the patient agreed to undergo a cementless left shoulder reverse arthroplasty.

Operative technique

**Figure 4:**
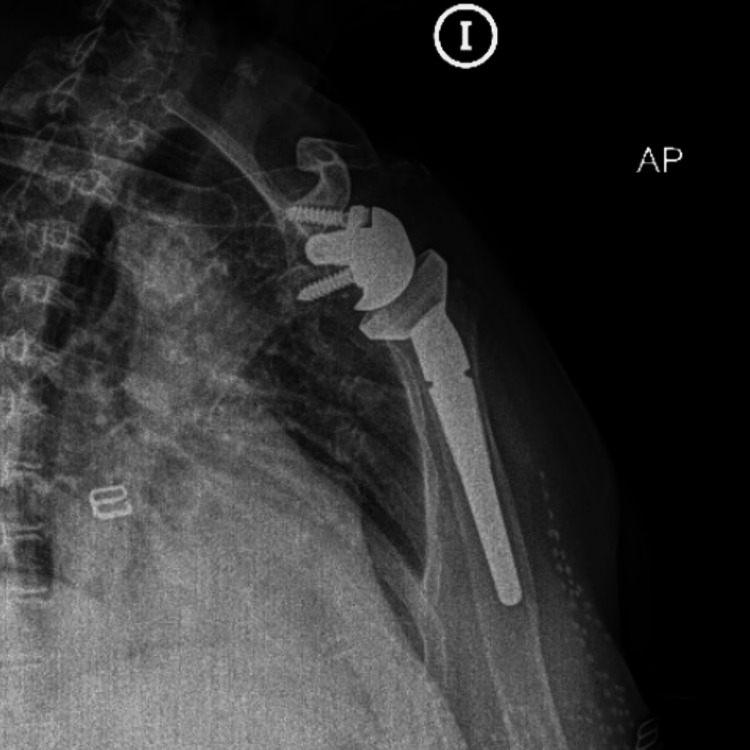
Post-surgical radiograph showing the presence of an uncemented reverse right shoulder prosthesis.

The surgery was performed under general anesthesia. A deltopectoral approach was used to expose the shoulder joint. The humeral head was removed, and the glenoid fossa was prepared for the prosthetic implant. A stem number 15 chrome-cobalt glenosphere with eccentric metaglene 36, reverse polyethylene insert 36 was implanted. The rotator cuff was not repaired (Figure 4).

Postoperative management

Antibiotics were routinely used one day postoperatively. Shoulder Arthroplasty Outpatient Appropriateness Risk Calculator was 2/11.

Passive range of motion exercises of the shoulder was performed under the supervision of physiotherapists within 10 days after the operation. She started an active assisted movement of the shoulder with restriction of external rotation for six weeks.

Follow-up

At the third-month follow-up visit, she had regained 60 degrees of flexion, 60 degrees of abduction, and 15 degrees of external rotation. Internal rotation was abolished. She achieved 11 points at the American Shoulder and Elbow Surgeons (ASES) score, 80 points at the Rowe score, 77.1 University of California Los Angeles (UCLA) shoulder score, and Shoulder Arthroplasty: Discharge Location Risk Calculator: prediction 0.01891.

## Discussion

Inveterate dislocation is a shoulder dislocation that has failed to respond to conservative treatment. It is defined as a dislocation that has been present for more than three weeks [6]. Inveterate dislocations are more common in elderly patients and patients with poor bone quality [6]. When conservative treatment for shoulder dislocation fails, it can be due to soft tissue and bone damage. The anterior soft tissues can stretch, and the posterior capsule and cuff can contract. This can lead to cartilage damage [11]. In our patient, she had a left shoulder dislocation that had been present for four months. She had a Hill-Sachs lesion, which is a compression injury of the humeral head, in a 40% which caused recurrent instability [12]. Hence, the main reason for complications in our patient was intense pain (9/10 VAS) and limited range of motion.

It is well known that osteonecrosis most commonly affects the superior middle portion of the humeral head. This is because the humeral head receives its blood supply from three main arteries: the ascending branch of the anterior humeral circumflex artery, the posterior humeral circumflex artery, and the arcuate artery. The ascending branch of the anterior humeral circumflex artery is the largest of these arteries and provides most of the blood supply to the humeral head. The posterior humeral circumflex artery enters the humeral head through the bicipital groove and pierces the rotator cuff attachments, providing a small amount of collateral flow. The arcuate artery arises from the posterior humeral circumflex artery and provides blood supply to the inner part of the humeral head. If the blood supply to the humeral head is interrupted, it can lead to cell death, joint collapse, pain, and loss of function [5].

The diagnosis of osteonecrosis is made based on both clinical and radiographic findings. Radiographs can be used to help diagnose and stage osteonecrosis of the humeral head. The most common views obtained are anteroposterior (AP), true AP, and axillary. MRI is the imaging study of choice when there is high clinical suspicion of osteonecrosis but no evidence of it is found on radiographs. MRI can also be used to predict the future collapse of the humeral head [13].

In our case, the patient's X-rays showed signs of stage III aseptic osteonecrosis of the humeral head, according to the modified Ficat classification [14]. This stage is characterized by the presence of a "crescent sign," which is a subchondral lucency area in the humeral head that indicates subchondral fracture and subsequent attempts at repair at the X-rays and the MRI [14]. Radiological evaluation is important for the diagnosis of osteonecrosis of the humeral head because patients with this condition do not typically develop symptoms as early as patients with osteonecrosis of the hip.

The Goutallier classification is a method for evaluating the extent of fatty degeneration in the rotator cuff muscles. It was originally developed for use with CT scans of the shoulder, but it can also be applied to MRI scans. The classification is based on the percentage of muscle atrophy and fatty degeneration, with grades 1-3 being used. Higher grades are associated with worse outcomes following surgical repair of a rotator cuff tear [13-15]. Our patient was classified as grade 3, which means 50% fatty muscle atrophy.

Other imaging studies that can be used to diagnose osteonecrosis of the humeral head include bone scintigraphy. However, this study is not commonly used due to the superiority of MRI and CT as they can provide more detailed information about the extent of the disease [15].

There are several classification systems used to describe avascular necrosis of the humeral head. The most common systems are the Ficat and Arlet system, the Steinberg system, the Association Research Circulation Osseous (ARCO) system, and the Japanese Investigation Committee (JIC) system [14,16]. Table 1 shows the classifications and findings in the different imaging studies by stages.

**Table 1:** Classifications of osteonecrosis ARCO: Association Research Circulation Osseous

	Stage					
Classification	0	I	II	III	IV	V
Ficat and Arlet	Radiograph: normal MRI: normal	Radiograph: normal or minor osteopenia MRI: edema	Radiograph: mixed osteopenia and/or sclerosis and/or subchondral cysts, without any subchondral lucency (crescent sign) MRI: geographic defect	Radiograph: crescent sign and eventual cortical collapse MRI: same as plain radiograph	Radiograph: end-stage with evidence of secondary degenerative change MRI: same as plain radiograph	
The ARCO classification	Radiograph: normal MRI: normal	Radiograph: normal MRI or bone scan: abnormal	Radiograph: trabecular bone changes without changes in subchondral bone; preserved joint space MRI: abnormal, diagnostic appearance	Presence of trabecular bone changes and subchondral fracture (crescent sign or subchondral bone collapse); preserved joint space Further subdivided with regard to the depth of femoral/Humerus head depression: IIIA - depression ≤2 mm; IIIB - depression >2 mm	Features of osteoarthritis, with the distorted femoral head shape, acetabular changes, and narrowed joint space	
Steinberg	Radiograph: normal MRI: normal	Radiograph: The appearance may vary from regular to subtle trabecular mottling MRI: shows abnormal bone.	- Stage IIa - focal radiopacity is associated with osteopenia; -Stage IIb radiopacity is associated with osteoporosis and an early crescent sign.	IIIa - an established crescent sign is associated with cyst formation IIIb - a subchondral fracture causes mild alteration in the configuration of the femoral/humeral head, and joint space is maintained.	The marked collapse of the femoral/humeral head is demonstrated	Joint space narrowing is demonstrated with changes in secondary osteoarthrosis.

The UCLA score is a system used to assess the function of the shoulder joint. It is based on three parameters: pain on active anterior flexion of the shoulder, patient satisfaction, and anterior flexion strength and function. The maximum possible score is 35, and a score of 27 or higher is considered excellent. A score of less than 27 is considered poor [17]. In our case, the patient achieved a UCLA score of 77.1. This indicates that the patient had good function of their shoulder joint. Another score used to assess joint function in this study was the Rowe score. The Rowe score is a system used to assess the outcome of surgery for shoulder instability. It is based on four factors: function, pain, stability, and motion. The score ranges from 0 to 100, with a higher score indicating a better outcome [17]. The patient scored 80 points on the follow-up exam, which indicates that their shoulder function had improved after treatment.

The treatment of osteonecrosis of the humeral head is complex and depends on the stage of the disease, the patient's age, and their activity level. There are a variety of treatment options available. They range from non-surgical or conservative treatment to surgical options such as central decompression, total shoulder arthroplasty, or reverse total shoulder arthroplasty (RTSA). The latter is considered the treatment of choice in the late stages (3, 4, or 5) of the disease [18], as was the case with the patient mentioned.

Shoulder arthroplasty can correct abnormal glenoid morphology by reshaping the socket so that the humeral head fits snugly inside it. The choice to use hemiarthroplasty or total arthroplasty is typically based on the glenoid status and the surgeon's opinion. The RSA is a type of shoulder arthroplasty that has become a popular therapy option for patients with arthropathy and rotator cuff damage [3]. In RSA, the rotation center of the shoulder is moved medially. This helps to reduce the amount of stress on the rotator cuff and allows the deltoid to raise the arm more effectively [19]. The deltoid is the largest muscle in the shoulder and is responsible for raising the arm. In RSA, the deltoid is used to lift the arm instead of the rotator cuff.

The merits of RSA are improved pain relief, improved function, and long-term success because studies have shown that the majority of people who have undergone the procedure are satisfied with the results and that the improvement in pain and function is sustained over time [8-11]. Nevertheless, the demerits of reverse shoulder arthroplasty are the risk of complications such as infection, nerve damage, and loosening of the implants; also, the limited range of motion as RSA does not restore the full range of motion of the shoulder joint. People who have undergone the procedure may have some limitations in their ability to rotate the arm internally and externally.

The indications for an RSA are those that compromise the function of the shoulder joint. They include rotator cuff tear arthropathy, irreparable massive rotator cuff tear, arthritis (primary osteoarthritis, rheumatoid arthritis), revision of failed total shoulder arthroplasty, severe shoulder joint instability, osteonecrosis of the humeral head. The patient had two of the indications for RTSA: shoulder joint instability and osteonecrosis. The patient had successful results with RTSA, which relieved their pain and improved their function [8-11,19].

RSA is a surgical procedure that has been developed in recent years to treat a variety of conditions that affect the shoulder joint. Osteonecrosis is a condition in which the blood supply to the humeral head is reduced or cut off. This can cause the humeral head to collapse and the shoulder joint to become painful and stiff. RSA is a viable treatment option for patients with osteonecrosis who have failed other treatments [8,9,12,19], such as non-surgical management or total shoulder arthroplasty.

In a study published in the Journal of Shoulder and Elbow Surgery, researchers found that RSA was effective in relieving pain and improving function in patients with osteonecrosis of the humeral head. The study included 20 patients who underwent RSA and were followed for an average of two years. The researchers found that the patients had significant improvements in pain, function, and quality of life after surgery [20]. The researchers concluded that RSA is a safe and effective treatment option for patients with osteonecrosis of the humeral head who have failed other treatments. They noted that RSA can provide significant pain relief and improve function in the shoulder joint.

Updating the evaluation of imaging studies can help to perform an adequate preoperative evaluation and have good postoperative results. The results of the six-month postoperative follow-up showed significant improvement in the VAS score, ASES score, UCLA score, and Rowe score, as well as in the functional status of the shoulder joint. The improvement in these scores indicates that the patients had a significant improvement in their shoulder pain, function, stability, and overall outcome after six months of surgery.

However, there are a few limitations of our study. First, we did not use the Constant-Murley score either initially or at follow-up. Second, the follow-up period of this study was short. Despite these limitations, the study provides promising evidence that RSA may be an effective treatment option for patients with osteonecrosis of the humeral head. Additional research is needed to confirm these findings and to determine the long-term effects of RSA on patients with osteonecrosis.

## Conclusions

Osteonecrosis of the humeral head after a glenohumeral dislocation is a rare condition. In the early stages, non-surgical conservative treatment may be effective. However, in the late stages, surgery may be necessary. Reverse shoulder arthroplasty is a surgical procedure that can be effective in relieving pain and improving function in patients with osteonecrosis of the humeral head. In this case, the patient had a successful reverse shoulder arthroplasty and reported significant improvements in recovery, range of motion, and quality of life. The patient was able to return to their previous activities without pain and was very satisfied with the results of the surgery.
